# Hospital Festivals and Meetings

**Published:** 1895-03-02

**Authors:** 


### HOSPITAL FESTIVALS AND MEETINGS.

### CHARING CROSS HOSPITAL.

The annual general court of the governors of the above hospital was held on February 20th in the board-room, Mr. John B. Martin (the treasurer) presiding. The report submitted by Mr. Arthur E. Reade, the secretary, showed that the council found themselves almost in the same position as on December 31st, 1893, and had the gratification of being able to report that the work of the hospital had been carried on without stint or contraction during the past twelve months. The most memorable event of the year was the laying of the foundation-stone of the convalescent home at Limpsfield Common, near Oxted, Surrey, on July 12th, by a generous donor, Mr. J. Passmore Edwards. It is hoped this may be completed during the present year. The number of in-patients during the year was 2,049, and of out-patients 23,696, making a total of 25,745 for the year. The ordinary income for the year amounted to £11,869, against £8,456 last year. The donations had risen from £2,472 to £5,684, and annual subscriptions from £1,908 to £1,916, whilst the results of donation boxes had fallen from £183 to £164. The grant from the Hospital Sunday Fund was £1,006 5s., against £875, whilst £241 9s. had been received from the Hospital Saturday Fund, against £238 in the previous year. The amount received in legacies was £971 over the previous year's receipts from the same source. The report concluded by an urgent appeal from the council on behalf of the institution. It was pointed out that the expenditure of the hospital amounted to £14,000, while the regular income, including annual subscriptions, amounted at the most to £4,000. There was an amount of £10,000 already due to the bankers, and that deficit tended constantly to increase. The report was adopted, and the proceedings closed with the usual votes of thanks.

### KING'S COLLEGE HOSPITAL.

The annual court of the Corporation of King's College Hospital was held on February 20th in the board-room of the hospital, Mr. George W. Bell in the chair. The Rev. Dr. Wace read the report, which stated that the hospital had been kept in full work. The total number of patients treated during 1894 was 2,468; the daily average of beds occupied, except in the vacation, was 192 (somewhat below the previous year); and the total number of out-patients' attendances was 22,646, which was above the average. The committee felt it would be very lamentable if, as was unhappily the case a few years ago, the work had to be curtailed. The ordinary expenses of the hospital during the year were £19,430, as compared with £19,339 in 1893. The annual subscription had increased from £2,266 to £2,357, but the ordinary donations showed a decrease from £4,306 to £3,862. The five years' maintenance fund, which was set on foot in 1893 yielded £2,700 in 1894. Altogether the ordinary receipts amounted to £14,250, leaving a deficit of over £6,000, to meet which the committee were obliged to use the legacies which accrued during the year, amounting to £6,259. At the festival dinner, presided over by Lord Davey, the sum of £2,449 was collected. The rearrangement recently made in the working hours of the nursing staff had proved most beneficial to their health. Each nurse had now four hours' recreation every day, and there had been a striking diminution in the sick-list. At the present time, out of eighty-seven there was only one ill, and that from a cause independent of her nursing work. The usual thanks were accorded to the medical and other officers, and a vote of thanks to the chairman closed the proceedings.

### THE SEAMEN'S HOSPITAL SOCIETY, GREENWICH.

The seventy-fourth annual court of this society was held in Sion College on the afternoon of the 20th inst. Lord Brassey, who was accompanied by Lady Brassey, presided; and there were also present the Bishop of Colchester, M. de Bille (Danish Minister), Sir J. Whittaker Ellis, Admiral de Kantzow, Admiral Egerton, Sir Frederick Young, Mr. P. A. Nairne, and Mr. Silas Waymouth, R.N.

The annual report, as submitted by Mr. Michelli (the secretary), showed that during the year no fewer than 17,427 patients had applied for relief at the Dreadnought Hospital, Greenwich, the Royal Victoria and Albert Dock Branch Hospital, and at the two dispensaries. During the year the deficiency in the funds, which for the previous three years had been over £2,000 annually, had been reduced to £1,411. The committee had, after careful consideration, decided that it would not be advisable in the meantime to erect a branch hospital at Tilbury, as had been proposed. While acknowledging with gratitude the support they had received during the year, the committee earnestly begged once more for further assistance to enable them to continue the work of tending sick and injured seamen in the port of London.

Lord Brassey moved the adoption of the report. Seamen, he said, were a class to whom the nation were most deeply indebted, and it ought not to be difficult to plead in London for a seamen's institution. That society and its hospitals supplied an urgently felt want; but it was matter for regret that the work was done at considerable pecuniary sacrifice. A deficit had occurred solely because it had been found necessary to establish a branch hospital at the Royal Victoria and Albert Docks, where, during the past year, 300 in and over 5,000 out-patients had been treated. He would appeal to the public for funds to supply means to increase the number of branches; and he would also appeal to ladies to follow the good example of his wife—(hear, hear)—in regularly visiting the hospitals.

The Bishop of Colchester, who seconded, appealed for aid on the ground that that was an old and well-tried charity. People were too much disposed to give all their support to new charities—the old should have the first claim.

Sir John Whittaker Ellis expressed the hope that the City companies would assist to clear off the deficit. (Applause.)

M. de Bille, Danish Minister, who was received with applause, moved the re election of the office bearers. He wished to pay a tribute to the international character of the society—to express the gratitude of foreign nations for what had been done for their sailors. (Applause.)

Mr. P. A. Nairne seconded.

To Lord and Lady Brassey, on the call of Sir Frederick Young and Admiral Egerton, a hearty vote of thanks was passed. Both speakers referred in cordial terms to the good work that Lady Brassey had done, and conveyed to Lord Brassey the best wishes of the meeting in connection with his journey to the Antipodes.

### QUEEN CHARLOTTE'S LYING-IN HOSPITAL.

The annual general meeting of the governors and subscribers of this charity was held at the hospital on Monday, February 25th, the chair being taken by Major McMahon, in the absence of Viscount Portman, the president.

The report, which was duly adopted, showed that a larger number of patients than ever before had benefited by the charity during 1894, the numbers being 1,079 in-patients, and 1,318 out-patients. The income fell short of the expenditure to the amount of £1,570 5s. 6d., to meet which deficiency it was necessary to sell £1,500 stock. Increased grants were received from the Hospital Sunday and Saturday Funds. Donations and subscriptions both unfortunately showed a decrease upon the previous year. A donation of £300 had been sent by Baron de Hirsch, and many useful gifts in kind from other friends had been received. During the year H.R.H. the Duchess of York consented to become a vice patron of the hospital, the secretary (Mr. G. Owen Ryan) resigned, and Mr. Arthur Watts was elected in his place. The progress in the midwifery training school has been satisfactory, the total number of medical students and pupils in midwifery and monthly nursing having increased by over 30 per cent. Miss M. F. McCord had been appointed matron, in succession to Mrs. Phillips, resigned. Help is urgently needed to carry on the work of Queen Charlotte's Convalescent Home, Kilburn, which was founded and maintained for many years by Mrs. Charles Roundell, but for which the committee are now responsible. It is a most important adjunct to the hospital, and supplies a much needed want in providing a temporary refuge for those mothers who are not strong enough to return straight home from the hospital. Certain structural additions and alterations in the hospital building are found to be necessary in order to meet the increased demands upon the accommodation. It is also probable that during the present year the premises in Marylebone Road, now occupied by the nursing staff, will be required by the Manchester, Sheffield, and Lincolnshire Railway. The committee are now considering the steps to be taken in regard to these changes.

### THE ROYAL SEA BATHING INFIRMARY FOR SCROFULA.—FESTIVAL DINNER.

A festival dinner in aid of the funds of the Royal Sea Bathing Infirmary at Margate was held in the Hotel Metropole on the evening of the 20th inst. A special appeal has been made for funds for this charity, which deserves to be more widely known and supported than it has been. Founded in 1791, it has for over a century rendered invaluable service in relieving and curing the scrofulous. The sea breezes of Margate are by some regarded as almost specific for scrofula, and no doubt the hospital owes its success in great part to its situation. Moreover, thanks to the generosity of the late Sir Erasmus Wilson and others, the buildings meet every requirement in connection with the treatment of scrofula in its various forms. It contains 220 beds. Unfortunately, however, the charity has of late years suffered severely in its finances; so much so that in 1892 it became necessary to close 140 of the beds—eighty only being left. But since then, through the generosity of an anonymous benefactor, ten more beds have been opened—that is to say, that ninety patients can be received in a hospital which has accommodation for 220.

The friends of the institution, who have resolved to remedy this state of matters, have reason to be gratified with the success of the festival dinner. There was a heartiness about the proceedings that has already had good results. The Lord Chancellor, who presided, made an excellent chairman. The guests, who numbered about fifty, included Earl Stanhope, Sir Russell Reynolds, Sir Dyce Duckworth, Sir Trevor Lawrence, Sir Frederick Wigan, Sir Richard Wyatt, the Rev. Prebendary Whittington, Mr. Timothy Holmes, Mr. Biddulph, M.P., and Mr. A. G. H. Gibbs, M.P. The toast list was brief, but the speeches kept to the point, and were permeated by a sincere belief in the good work that is done by the hospital.

The LORD CHANCELLOR, who proposed the toast of the evening, had no difficulty in making out a good case for his client. If funds had fallen off it was not because the hospital was less needed; nor had it become less efficient. The patients who had been discharged "fully cured"—a large proportion, indeed—were conclusive evidence as to the work done. (Applause.) The diseases to which the hospital ministered were of a specially painful character; it was distressing to think how many might have been relieved if it had not been necessary to close these beds. At that hospital patients were received from all parts of the country, but it was worthy of note that more than half of them came from London; indeed, many were sent from London hospitals. To the medical profession the hospital was greatly indebted, and in particular he would refer to Sir. John Whitaker Hulke, president of the Royal College of Surgeons, whose death they so deeply regretted—a man of remarkable modesty, whose upright character was respected by all who knew him. (Applause.) The Lord Chancellor trusted that the friends of the hospital would do their best to make it known—in these days of advertising that was necessary; and that they would not rest content until every bed was open again. (Applause.)

Mr. MICHAEL BIDDULPH, M.P., treasurer, in replying, mentioned some of the causes that had brought about the falling off in their funds. The principal cause, in his opinion, was the unusual and prolonged depression in agriculture. Although the improved methods in the treatment of scrofula had yielded much better results than the old, it should not be forgotten that the expense of treatment was now much greater than it used to be. That was the only hospital for scrofula in the United Kingdom; it was therefore really a national institution.

Rev. Prebendary WHITTINGTON gave some interesting particulars as to the history of the hospital. He trusted that they would be able to open at least ten more beds within a very short time.

Sir RUSSELL REYNOLDS, who was heartily received, endorsed the claim that had been put forward for the air of Margate. There was no doubt there was something in the air of Margate that did good in cases of scrofula. His own feeling was that the place was not a very attractive one—(laughter)—still, if he had scrofulous disease, he would certainly go there. He had been down seeing the hospital, and it was most distressing to find so many beds closed. He thought that the institution was not sufficiently well known.

Sir RICHARD VERRALL proposed the toast of "The Lord Chancellor," which was cordially pledged; and ere the company broke up, subscriptions and donations to the charity of over £1,050 were reported—an announcement which was received with cheers.

